# Voluntary Wheel Running Reverses Deficits in Social Behavior Induced by Chronic Social Defeat Stress in Mice: Involvement of the Dopamine System

**DOI:** 10.3389/fnins.2019.00256

**Published:** 2019-04-04

**Authors:** Jing Zhang, Zhi-xiong He, Li-min Wang, Wei Yuan, Lai-fu Li, Wen-juan Hou, Yang Yang, Qian-qian Guo, Xue-ni Zhang, Wen-qi Cai, Shu-cheng An, Fa-dao Tai

**Affiliations:** Institute of Brain and Behavioral Sciences, College of Life Sciences, Shaanxi Normal University, Xi’an, China

**Keywords:** voluntary wheel running, chronic social defeat stress, dopamine system, D2 receptors, nucleus accumbens

## Abstract

Voluntary exercise has been reported to have a therapeutic effect on many psychiatric disorders and social stress is known to impair social interaction. However, whether voluntary exercise could reverse deficits in social behaviors induced by chronic social defeat stress (CSDS) and the underlying mechanism remain unclear. The present study shows CSDS impaired social preference and induced social interaction deficiency in susceptible mice. Voluntary wheel running (VWR) reversed these effects. In addition, CSDS decreased the levels of tyrosine hydroxylase in the ventral tegmental area and the D2 receptor (D2R) in the nucleus accumbens (NAc) shell. These changes can be recovered by VWR. Furthermore, the recovery effect of VWR on deficits in social behaviors in CSDS mice was blocked by the microinjection of D2R antagonist raclopride into the NAc shell. Thus, these results suggest that the mechanism underlying CSDS-induced social interaction disorder might be caused by an alteration of the dopamine system. VWR may be a novel means to treat CSDS-induced deficits in social behaviors via modifying the dopamine system.

## Introduction

Social interaction disorders such as social avoidance and lack of desire for social interaction are common symptoms of psychiatric disorders (Miyoshi and Morimura, 2010; Rosa et al., 2018). Social interaction disorders can be induced by social stress such as bullying and workplace harassment (Bonafons et al., 2009; Halabi et al., 2018). Chronic social defeat stress (CSDS) is widely used for research on the social stress in animal models (Krishnan et al., 2007; Rosa et al., 2018). In general, mice show a tendency to investigate novel same-sex conspecifics (Murugan et al., 2017). Previous reports showed that CSDS can reduce the tendency to interact with other individuals in susceptible mice, and symptoms include social avoidance and decreased social sniffing (Jin et al., 2015; Huang et al., 2016). This effect can last at least 3 weeks after the last day of CSDS (Krishnan et al., 2007; Razzoli et al., 2011). However, the mechanisms in the brain underlying the changes induced by CSDS in these social behaviors are currently poorly understood. An improved understanding of these mechanisms is critical in finding novel treatment options for these social interaction disorders.

Growing evidence shows that the dysfunction of mesolimbic dopamine (DA) neurons negatively impacts behavioral responses to social stress (Trainor, 2011). The mesolimbic DA pathway is composed of several brain regions, including the ventral tegmental area (VTA), the nucleus accumbens (NAc), and the medial prefrontal cortex (mPFC). The VTA and its projections to NAc allow an organism to identify and learn about outcomes that are associated with stimuli, as well as to initiate appropriate approaches or avoidance responses (Wise, 2004). CSDS decreases social sniffing in susceptible mice in social interaction tests, which might involve a dysfunction of the DA receptor (Jin et al., 2015; Huang et al., 2016). In addition, injection of DA can rescue such deficits in social interaction in Dcf1 (dendritic cell factor 1, also known as TMEM59) mice, which are gene knockout mice with social interaction disorder (Liu et al., 2017). Thus, we hypothesize that CSDS-induced social interaction disorder might involve alterations in the DA system.

The NAc is a major projection site of dopaminergic neurons, which contain high levels of D1 and D2 dopamine receptors (D1R and D2R). Recent findings show that D2R in the NAc core increases only when exposed to social defeat stress in adolescent rats; however, no significant D2R alteration was observed in the same region in adolescent rats exposed to foot-shock stress (Burke et al., 2011). A further report showed that chronic passive exposure to aggression decreases D2R densities in the NAc shell in rats (Suzuki et al., 2010). In addition, only a significant decrease of D1R was observed in the prefrontal cortex (PFC) in CSDS mice, while no significant difference of D2R was observed (Huang et al., 2016). Another study reported that CSDS exerts no significant effects on D1R and D2R expression in PFC in CSDS mice (Jin et al., 2015). Thus, effects of CSDS on the levels of D1R and D2R are region-specific and stress-type-dependent. More studies are required to investigate the effects of CSDS on levels of D1R and D2R in different brain regions.

Clinical studies showed that exercise has significant effects in ameliorating psychiatric disorders, the efficacy of which is as good as that of the drug sertraline, which is used to treat psychiatric disorders. Moreover, exercise treatments have lower recurrence rates of psychiatric disorders and side effects than sertraline (Blumenthal et al., 2007; Hoffman et al., 2011). Voluntary wheel running (VWR) is a type of voluntary exercise that is regularly used in pre-clinical studies. VWR is a natural exercise intervention (Mul, 2018), similar to human motivation to exercise. A recent report showed that VWR reverses high levels of defensive/submissive behaviors induced by a single social defeat stress in Syrian hamsters (Kingston et al., 2018). Otsuka et al. pointed out that voluntary running on a wheel for 2 h can reduce the social avoidance behavior induced by CSDS (Otsuka et al., 2015). However, whether brain monoamine levels are altered by VWR remains unclear. The activation of DA receptors was found to contribute to VWR (Correa et al., 2016; Zhu et al., 2016). Thus, whether VWR can treat social interaction disorder induced by CSDS remains largely unknown. Furthermore, it remains unclear whether VWR ameliorates social interaction disorder via alteration of the DA system.

Hence, this study first examined the effects of VWR on CSDS-induced alterations of the social behavior and DA system in mice. Second, this study investigated whether VWR can reverse the CSDS-induced alteration of the DA system. Lastly, this study explored whether DA receptors play an essential role in the reversal of CSDS-induced alteration of social interaction via VWR.

## Materials and Methods

### Animals

Healthy male C57BL/6J mice (7–8 week) and retired breeding mice of CD1 male mice (6 months) were purchased from the Laboratory Animal Breeding and Research Center of The Fourth Military Medical University and from Xi’an Jiaotong University (Xi’an, China), respectively. Mice were fed in a controlled room (Temperature: 22 ± 2°C, Humidity: 60 ± 5%, 12 h/12 h: light/dark cycle, 06:30/18:30) with access to food and water *ad libitum*. All procedures were approved by the Animal Care and Use Committee of Shaanxi Normal University and were in accordance with the Guide for the Care and Use of Laboratory Animals of China.

### CSDS Paradigm

**FIGURE 1:**
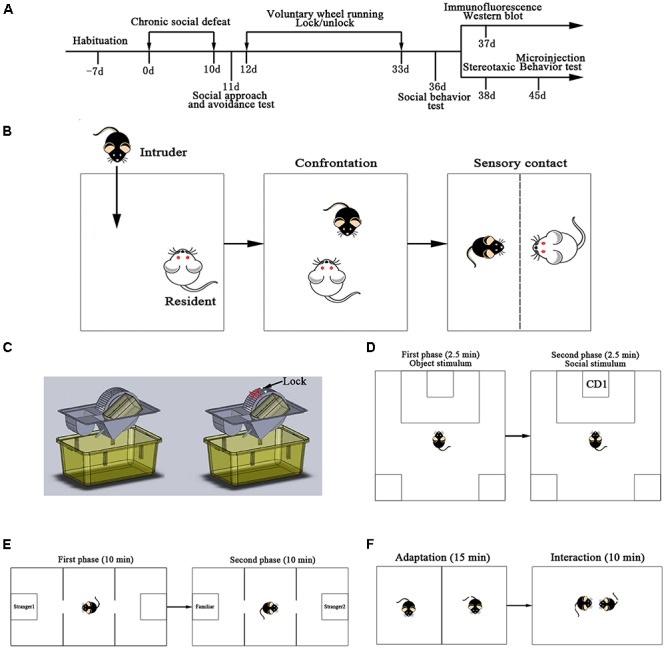
Experiment design. **(A)** Time-line showing all the steps of the experimental manipulations. **(B)** Resident-intruder paradigm was used as chronic social defeat stress. **(C)** Voluntary wheel running was used in this study. **(D)** Scheme of the social approach and avoidance test. **(E)** Scheme of the three-chambered social test. **(F)** Scheme of the social interaction test.

After 7 days of acclimation, C57BL/6J mice were randomly assigned to two groups: control group (NC, *n* = 12) and CSDS group (SD, *n* = 38). Mice from the SD group underwent a repeated social defeat stress paradigm (Figure 1A,B). The resident-intruder social stress paradigm was applied as previously reported with a slight modification (Golden et al., 2011). CD1 mice were selected on the basis of their attack latency (shorter than 30 s on three consecutive screening tests) and were classified as aggressive residents. The resident CD1 mouse remained solely in its own home cage. A C57BL/6J mouse as an intruder was exposed to a strange resident CD1 mouse. After 10 min of confrontation, they were separated via holed clear perforated acrylic glass (allowing the animals to see, hear, and smell each other, but preventing physical contact). They were exposed to chronic stress in the form of this threat for the next 24 h. In case the CD1 severely attacked the C57BL/6J, the defeat bout was immediately interrupted by the experimenter (Figure 1B). After 24 h, the intruder mouse was exposed to another strange resident CD1 mouse. The paradigm was consistently conducted between 2 pm and 5 pm for 10 days. Control group mice were pair-housed in similar cages and took turns in different cages every day; however, these were not treated to CSDS.

### Social Avoidance Test

The social approach and avoidance behaviors of mice in the SD group (*n* = 38) were observed 1 day after CSDS. Before the tests, experimental mice were placed into the behavioral testing room for at least 1 h. The social approach and avoidance test consisted of a two-trial procedure under dimly lit conditions: during the first 2.5 min of the test (target absent condition), the experimental mouse was allowed to freely explore a square shaped open filed arena (48 × 48 cm) containing a mesh cage (7 cm × 7 cm × 14 cm high) placed on one side of the interactive arena. The experimental C57BL/6J mouse was then removed from the testing arena for 1 min and placed into a separate cage. In the second 2.5 min of the test (target present condition), the experimental mouse was reintroduced into the arena, which contained a novel CD1 mouse in a mesh cage. “Interaction Zone” (16 × 28.8 cm) and “Corner Zone” (9.6 × 9.6 cm) were assigned as previously reported with a slight modification (Golden et al., 2011; Figure 1D). The duration was also recorded and scored using an automated video tracking system (Shanghai Xinruan Information Technology Co., Ltd., China). The interaction ratio was calculated as (interaction time, target present)/(interaction time, target absent) and normalized to 100. Susceptible and unsusceptible mice were separated based on the interaction ratio: mice with scores <100 were defined as “susceptible” and those with scores ≥100 were defined as “unsusceptible” or resilient (Chaudhury et al., 2013). Between sessions, the box was cleaned with 70% ethanol and dried with paper towels. From the 38 mice exposed to CSDS, 24 were susceptible and 14 were unsusceptible. Susceptible animals were used for the following study.

### Exercise Protocol

To test the effects of voluntary exercise on behavioral alteration induced by CSDS, susceptible animals were separated into SD group and SD + VWR group. Mice from the SD group were CSDS mice that were exposed to a locked wheel for 21 days. The mice from the SD + VWR group were CSDS mice that were constantly exposed to VWR for 21 days (Figure 1A,C). Control group mice were normally fed for 21 days. Exercise training was performed using a wheel with a diameter of 160 mm, corresponding to 0.5 m per revolution (Shanghai minlylab hi-tech development, Shanghai, China).

### Behavioral Test

#### Three-Chambered Social Test

After 21 days of VWR, the experimental mice were moved to the behavioral testing room for an adaptation of at least 1 h. The social test apparatus consisted of a box with three chambers (60 cm × 40 cm × 22 cm) under dimly lit conditions. First, the mouse was placed into the middle chamber and allowed to habituate for 10 min to three-chambers containing two empty mesh cages (7 cm × 7 cm × 14 cm) in each side-chamber. For the sociability test, an unfamiliar mouse (Stranger 1) was introduced into one of the mesh cages in one of the side chambers, leaving an empty mesh cage in the other side chamber. Then, the mouse was allowed to freely explore all three chambers for 10 min. Next, for the social preference test, a novel stranger mouse (Stranger 2) was introduced into the mesh cage that was previously empty, and the test mouse was again allowed to explore for 10 min (Figure 1E). The duration of the investigation was also recorded and scored using an automated video tracking system (Shanghai Xinruan Information Technology Co., Ltd., Shanghai, China).

#### Social Interaction Test

Behaviors of mice from different groups were observed during the social interaction test. The social interaction apparatus consisted of an open opaque acrylic box (37 × 27.5 × 18 cm). Each test mouse and an unfamiliar mouse of the same genetic background with similar weight and age were carefully and individually placed in the cage. They were separated via a paperboard to adapt to the new surroundings for 15 min. Then, the paperboard was removed and the following behaviors were recorded for 10 min using a digital video camera (Figure 1F): aggression, climbing (or mounting, crawling under or over), defensive (including escaping and surrender), exploration, freeze, and grooming. The total duration and frequency of these behaviors were scored using J Watch software^1^ by a researcher who was blind to the experimental design. All behavioral analyses have been described previously (Jia et al., 2009; Zeng et al., 2010; Huang et al., 2016; Wang et al., 2018).

### Immunofluorescence

Mice from different groups (*n* = 6 for each group) were anesthetized with pentobarbital sodium and transcardially perfused with 0.1 M PBS buffer (pH 7.4) followed by 4% paraformaldehyde. The brains were rapidly removed and immersed in 4% paraformaldehyde for 5–7 days, following 20% and 30% sucrose solution until saturated at 4°C. Afterward, the brains were sectioned at 30 μm thickness on a freezing microtome (CM1950, Leica, Germany) and used in the following experiment.

These slices were dried for 10 min at room temperature, then washed with 0.01 M PBS for 10 min, following incubation with 0.3% H_2_O_2_ for 20 min, and washed for 3 × 5 min with 0.01 M PBS. Next, sections were blocked in 5% BSA blocking solution (containing 0.2% Triton X-100, Boster, China) for 30 min, then preincubated for 60 min in blocking solution (normal goat serum, AR0009, Boster Company, China). All incubations were conducted in a dark humidifying box. After that, sections were incubated overnight at 4°C. Tyrosine hydroxylase (TH) immunofluorescence used purified rabbit polyclonal antibody of TH (1:500, ab112, Abcam, United States) diluted in antibody diluent (0.1 M PBS containing 60% bovine serum albumin). On the second day, sections were washed 3 × 5 min, and incubated in the goat anti-rabbit secondary antibody (SA1022, Boster Company, China) for 60 min in a dark humidifying box. All sections were visualized and images were captured with a fluorescent microscope (FV-1000, Olympus, Japan). Then, TH-immunoreactive (TH-ir) neurons in the VTA were bilaterally measured using the Image-Pro Plus software (V6.0, Media Cybernetics, United States).

### Western Blot Analysis

The mice of different groups (*n* = 6 for each group) were sacrificed with an overdose of sodium pentobarbital 24 h after the behavioral tests. The mPFC, core and shell regions of the NAc and VTA were bilaterally punched out using a 1.0 mm Harris Uni-Core micropuncher (Electron Microscopy Sciences, Hatfield, PA, United States) according to the mice brain atlases (Paxinos and Franklin, 2001). These regions were then stored at -80°C until further processing. Brain tissues were homogenized in ice-cold RIPA lysis buffer. Protein concentrations were quantified with the BCA protein assay kit (Thermo Fisher Scientific). Equal amounts of total protein were separated via SDS-PAGE on 8–12% polyacrylamide gels and transferred to a nitrocellulose membrane. The immunoblots were incubated with primary antibodies overnight at 4°C followed by incubation with the corresponding secondary antibodies (1:10000, Zhong Shan Golden bridge Biotechnology, China) at room temperature for 1 h. The blots were visualized with ECL-plus reagent and the results were quantified with a fully automatic chemiluminescence image analysis system (Tanon2000). Quantification was conducted using the Image Pro Plus software, and target protein data were normalized to GAPDH band. The following primary antibodies were used: D1R (1:500, ab81296, abcam), D2R (1:1000, ab85367, abcam, United States), TH (1:1000, ab112, abcam, United States), and GAPDH (1:8000, NC020, Zhuangzhibio, China).

### Stereotaxic Cannulation and Microinjection

The additional C57BL/6J mice (SD + VWR group, *n* = 24) received stereotaxic cannulation surgery following anesthesia with pentobarbital sodium combined with a mixture of isoflurane and oxygen under sterile conditions. 26-gauge stainless steel guide cannulae (RWD, China) were implanted bilaterally, aimed at the NAc shell (1.4 mm rostral, ± 0.5 mm bilateral, and -4.5 mm ventral to the bregma). Finally, the cannulae were affixed to the skull using dental cement. The D2R antagonist raclopride (abcam, United States) was diluted in sterile saline to obtain final concentrations of 5, 25, and 50 μg/μl. After 3 days of recovery, each mouse with normal activity (similar to that of animals without surgery) received microinjection of bilateral raclopride 1 μg/0.2 μl (*n* = 6), 5 μg/0.2 μl (*n* = 6), and 10 μg/0.2 μl (*n* = 6) or the same volume of saline (*n* = 6). The volume of injection was 0.1 μl for each NAc shell. The speed of injection was 0.1 μl/min and required about 1 min. The injection cannula was left in position for an additional 1 min after drug infusion. These doses were selected on the basis of previous studies with a slight modification (van den Boss et al., 1988; Miller and Lonstein, 2005; Benedetto et al., 2017; Steidl et al., 2017). The three-chambered social test and social interaction test were conducted within 30 min after microinjection. After the behavioral tests were completed, all subjects were anesthetized, and the brains were harvested. Then, the brains were cut into 30-μm sections on a cryostat to histologically verify the injection sites. The experimental data of animals in which the cannula tips were successfully located in the NAc shell were analyzed.

### Statistical Analyses

According to one-sample Kolmogorov-Smirnov tests, all data were normally distributed; therefore, parametric tests were used for in statistical analyses. The results are presented as mean ± SEM. Statistical analyses were performed using SPSS version 20.0 (SPSS Institute, Chicago, IL, United States). A paired sample *t*-test was used for analysis of data from sociality and social preference tests. Multivariate analysis of variance (ANOVA) was used to analyze the behavioral data of the social interaction test. One-way ANOVA was employed to evaluate the existence of differences among three or more groups. Once a significant difference was detected, Tukey’s multiple comparisons test was used to determine the significance between every two groups. A *p*-value <0.05 was considered statistically significant.

## Results

### VWR Improves CSDS-Induced Deficits in Social Preference and Social Interaction

**FIGURE 2:**
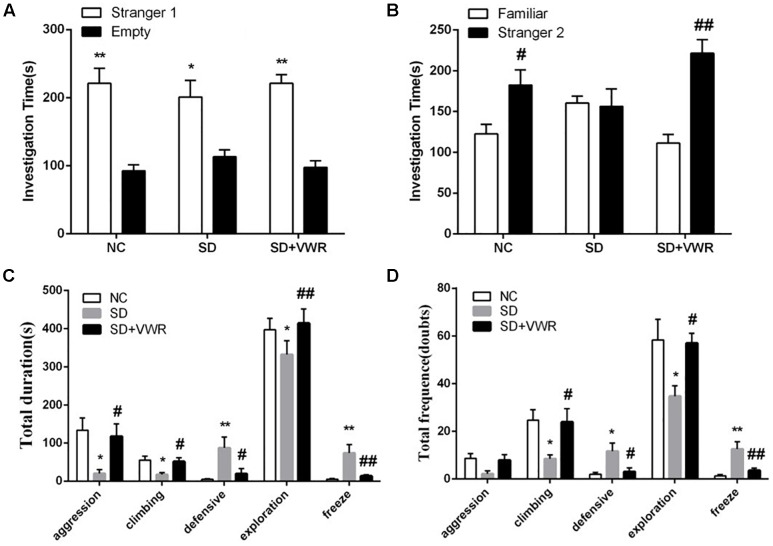
The effect of VWR on CSDS-induced alteration in sociability and social interaction. The sociability **(A)** and social preference **(B)** of mice were detected by three-chambered social test. **(A)** ^∗^*p* < 0.05 and ^∗∗^*p* < 0.01, stranger 1 vs. empty. **(B)** ^#^*p* < 0.05 and ^##^*p* < 0.01, stranger 2 vs. familiar. The total durations **(C)** and frequency **(D)** of social interaction behaviors were tested by social interaction test. *n* = 12. ^∗^*p* < 0.05 and ^∗∗^*p* < 0.01, vs. NC. ^#^*p* < 0.05 and ^##^*p* < 0.01, vs. SD. NC, control group; SD, chronic social defeat stress; SD + VWR, SD + voluntary wheel running.

The three-chambered social test was used to investigate the effect of VWR on the levels of sociability and social preference in CSDS-induced mice. Paired-samples *t*-test showed that the mice from all three groups spent more time investigating the chamber with the strange mouse than the empty chamber, showing similar levels of sociability (NC: *t* (11) = 4.384, *p* < 0.01; SD: *t* (11) = 2.724, *p* < 0.05; SD + VWR: *t* (11) = 6.938, *p* < 0.01) (Figure 2A). However, when faced with the choice of either entering a familiar chamber or a strange chamber, mice in the NC group and SD + VWR group spent less time investigating the chamber with familiar mice than the chamber with strange mice (NC: *t* (11) = -2.376, *p* < 0.05; SD + VWR: *t* (11) = -4.707, *p* < 0.01). However, there was no significant difference in the time spent by mice of the SD group in chambers with familiar mice and strange mice (*t* (11) = 0.157, *p* = 0.878), suggesting that VWR improved CSDS-induced deficiency of social preference (Figure 2B).

The social interaction test was used to evaluate the effect of VWR on social behaviors of CSDS mice. Multivariate ANOVA showed that the total durations and frequency of aggression (duration: *F_2_*_,_ *_33_* = 5.132, *p* < 0.05; frequency: *F_2,33_* = 3.436, *p* < 0.05), climbing (duration: *F_2,33_* = 5.351, *p* < 0.05; frequency: *F_2,33_* = 4.595, *p* < 0.05), defensive (duration: *F_2,33_* = 5.78, *p* < 0.01; frequency: *F_2,33_* = 5.768, *p* < 0.01), exploration (duration: *F_2,33_* = 6.424, *p* < 0.01; frequency: *F_2,33_* = 4.69, *p* < 0.05), and freeze (duration: *F_2,33_* = 8.078, *p* < 0.01; frequency: *F_2,33_* = 10.428, *p* < 0.01) were significantly different between groups. CSDS significantly decreased the duration of aggression (*p* < 0.05), climbing (*p* < 0.05), and exploration behavior (*p* < 0.05), while increasing the levels of defensive behavior (*p* < 0.01) and freeze (*p* < 0.01) compared to NC groups. VWR significantly reversed the effects of CSDS on aggression (*p* < 0.05), climbing (*p* < 0.05), exploration behavior (*p* < 0.01), defensive behavior (*p* < 0.05), and freeze (*p* < 0.01) compared to the SD group (Figure 2C). CSDS significantly decreased the frequency of climbing (*p* < 0.05) and exploration behavior (*p* < 0.05), while increasing the levels of defensive behavior (*p* < 0.05) and freeze (*p* < 0.01) compared to the NC group. VWR significantly reversed the effects of CSDS climbing (*p* < 0.05), exploration behavior (*p* < 0.05), defensive behavior (*p* < 0.05), and freeze (*p* < 0.01) compared to SD groups (Figure 2D). This result suggests that VWR improved CSDS-induced deficiency in social interaction.

### VWR Increases TH Levels in VTA of CSDS Mice

**FIGURE 3:**
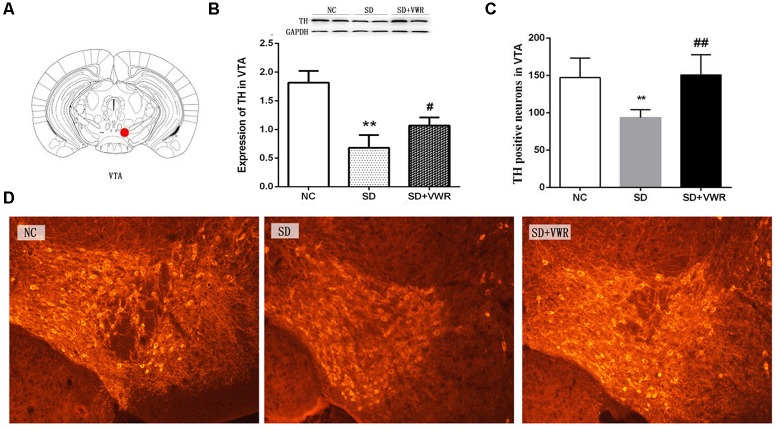
The effect of VWR on CSDS-induced changes in TH levels in the VTA. **(A)** Location of the VTA. **(B)** The expression of TH in the VTA was measured by western blot. **(C,D)** The numbers of TH positive neurons in the VTA was detected by immunofluorescence. Bar = 100 μm. *n* = 6. ^∗∗^*p* < 0.01, vs. NC. ^#^*p* < 0.05, and ^##^*p* < 0.01, vs. SD. TH, tyrosine hydroxylase; NC, control group; SD, chronic social defeat stress; SD + VWR, SD + voluntary wheel running.

Western blot analysis and immunofluorescence were used to investigate the effect of VWR on TH levels in CSDS mice. As shown in Figure 3, the expression of TH (*F_2,15_* = 8.722, *p* < 0.05) and the numbers of TH positive neurons (*F_2,15_* = 36.341, *p* < 0.01) in the VTA showed significant differences between the three groups. The expression of TH and the numbers of TH positive neurons in the VTA in the SD groups were significantly lower than those in the NC groups (expression of TH: *p* < 0.01, numbers of TH positive neurons: *p* < 0.01), and significantly lower than those in the SD + VWR groups (expression of TH: *p* < 0.05, numbers of TH positive neurons: *p* < 0.01) (Figure 3). These results indicate that TH in the VTA might be involved in the reverse effects of VWR on deficits in CSDS-induced social interaction.

### VWR Increases Expressions of D2R in NAc Shell of CSDS-Induced Mice

**FIGURE 4:**
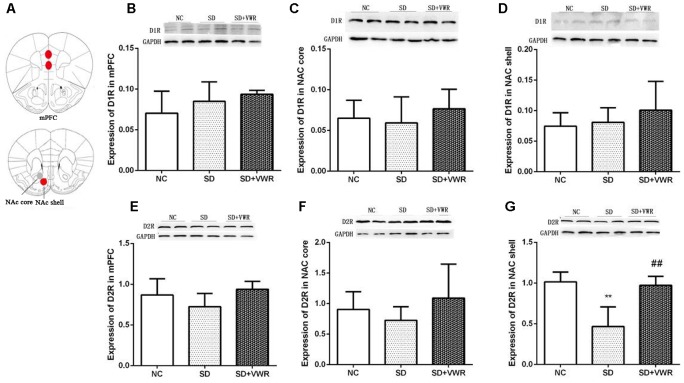
The effect of VWR on CSDS-induced changes in D1R and D2R levels in the mPFC and NAc. **(A)** Locations of the mPFC, NAc core and NAc shell. The expressions of D1R in the mPFC **(B)**, NAc core **(C)** and NAc shell **(D)** were measured by western blot. The expression levels of D2R in mPFC **(E)**, NAc core **(F)** and NAc shell **(G)** were measured by western blot. *n* = 6. ^∗∗^*p* < 0.01, vs. NC. ^##^*p* < 0.01, vs. SD. D1R, dopamine D1 receptor; D2R, dopamine D2 receptor; NC, control group; SD, chronic social defeat stress; SD + VWR, SD + voluntary wheel running.

Western blot analysis was used to evaluate the effect of VWR on expression levels of dopamine receptors in the mPFC and NAc of CSDS mice. As shown in Figure 4, expression levels of D1R in the mPFC (*F_2,15_* = 1.884, *p* = 0.754), NAc core (*F_2,15_* = 0.675, *p* = 0.505), and NAc shell (*F_2,15_* = 1.034, *p* = 0.561) were not significantly different among the three groups. Expressions of D2R in the mPFC (*F_2,15_* = 2.796, *p* = 0.083) and NAc core (*F_2,15_* = 0.61, *p* = 0.26) were not significantly different among the three groups. However, D2R levels in the NAc shell of mice in the SD group (*p* < 0.01) were significantly lower compared to the NC group, and VWR up-regulated CSDS-induced decrease in D2R expression in the NAc shell (*p* < 0.01) (Figure 4). These results suggest that D2R in the NAc shell might be involved in the reversal of the effects of VWR on deficits in social interaction induced by CSDS.

### Raclopride Induced D2R Blockages in the NAc Shell Induce Deficits in Social Interaction in Mice of the SD + VWR Group

**FIGURE 5:**
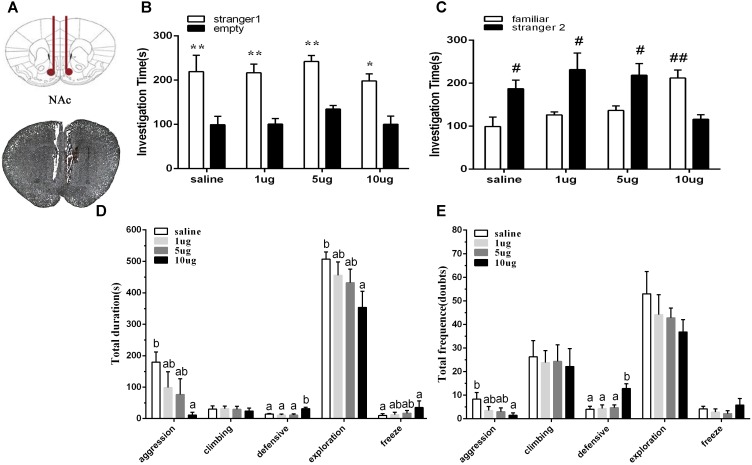
Effect of D2R blockage in the NAc shell on social behaviors in mice with SD + VWR. **(A)** Histological verification of injection sites located in the NAc shell. The sociability **(B)** and social preference **(C)** of mice were detected by three-chambered social test after microinjection of saline and 1, 5, 10 μg raclopride into the NAc shell. **(B)** ^∗^*p* < 0.05 and ^∗∗^*p* < 0.01, stranger 1 vs. empty. **(C)** ^#^*p* < 0.05 and ^##^*p* < 0.01, stranger 2 vs. familiar. The total durations **(D)** and frequency **(E)** of social interaction behaviors were tested by social interaction test after microinjection of saline and 1, 5, 10 μg raclopride into the NAc shell. *n* = 6. Groups not sharing the same letters are significantly different from each other in each behavior (*p* < 0.05). NC, control group; SD, chronic social defeat stress; SD + VWR, SD + voluntary wheel running.

To explore the involvement of D2R in the NAc shell on social behaviors in mice of the SD + VWR group, raclopride (a D2R specific antagonist) at dosages of 1, 5, and 10 μg were microinjected into the NAc of mice with SD + VWR via stereotaxic apparatus (Figure 5A). Then, the three-chambered social test and social interaction test were used to evaluate social behaviors of mice after D2R blockage. In the three-chambered social test, paired-samples *t*-test showed that mice spent more time investigating the chamber with strange mice than the empty chamber (saline: *t* (5) = 4.161, *p* < 0.01, 1 μg: (*t* (5) = 4.356, *p* < 0.01), 5 μg: (*t* (5) = 7.635, *p* < 0.01), and 10 μg (*t* (5) = 3.24, *p* < 0.05), showing similar levels of sociability among all four groups of mice (Figure 5B). However, when faced with the choice of entering a familiar chamber or a strange chamber, only mice microinjected with 10 μg of raclopride (*t* (5) = 5.795, *p* < 0.01) showed a preference for familiar mice, while other groups showed a significant preference for strange mice (saline: *t* (5) = -3.565, *p* < 0.05; 1 μg/μl: *t* (5) = -2.913, *p* < 0.05; 5 μg, *t* (5) = -2.767, *p* < 0.05) (Figure 5C). These results suggest that D2R blockage caused by 10 μg of raclopride induced a social preference deficit in mice with SD + VWR.

In the social interaction test, multivariate ANOVA showed that the total duration of aggression (*F_3,20_* = 3.159, *p* < 0.05), defensive behavior (*F_3,20_* = 8.234, *p* < 0.01), exploration (*F_3,20_* = 3.303, *p* < 0.05), and freeze (*F_3,20_* = 3.435, *p* < 0.05) were differed significantly among groups. No significant difference in the total duration of climbing was found (*F_3,20_* = 0.123, *p* = 0.946). Tukey *post hoc* tests showed that the 10 μg group spent less time engaging in aggression (*p* < 0.05), exploration (*p* < 0.05), and grooming (*p* < 0.05), but spent more time engaging with defensive behavior (*p* < 0.01) and freeze (*p* < 0.05) than the saline group. Mice of the 10 μg group spent more time engaging in defensive behavior than mice of the 1 μg group (*p* < 0.01) and the 5 μg group (*p* < 0.01) (Figure 5D). Multivariate ANOVA showed that the D2 receptors antagonists significantly changed the frequency of aggression (*F_3,20_* = 3.492, *p* < 0.05), defensive behavior (*F_3,20_* = 7.518, *p* < 0.01), and grooming (*F_3,20_* = 3.402, *p* < 0.05). Tukey *Post hoc* tests showed that mice of the 10 μg group exhibited a lower frequency of aggression (*p* < 0.05) and a higher frequency of defensive behavior (*p* < 0.01) than the saline group. Mice from the 10 μg group engaged in defensive behavior more frequently than those of the 1 μg group (*p* < 0.01) and the 5 μg group (*p* < 0.01). No significant differences were found in the total frequency of climbing (*F_3,20_* = 0.066, *p* = 0.978), exploration (*F_3,20_* = 0.593, *p* = 0.627), and freeze (*F_3,20_* = 0.829, *p* = 0.493) (Figure 5E). These data show that D2R blockage via 10 μg raclopride induced deficiency in social interaction in mice with SD + VWR.

## Discussion

In this study, VWR reversed the impairment of social preference and deficiency in social interaction induced by CSDS in susceptible mice. In addition, the decreased levels of TH in the VTA and D2R in the NAc shell induced by CSDS were also up-regulated by VWR. Furthermore, the recovery effect of VWR in CSDS mice with regard to deficits in social interaction was blocked by microinjection of the D2R antagonist raclopride into the NAc shell. Thus, this study provides a novel method to treat CSDS-induced social interaction disorder and verifies the involvement of the dopamine system in this process.

### VWR Reversed Impairment of Social Preference and Deficiency in Social Interaction Induced by CSDS

This study showed that CSDS impaired social preferences and induced deficiency in social interaction. In the social preference test, CSDS mice spent more time with familiar than novel mice. These results are consistent with a previous report where defeated mice exposed to aggressive CD1 mice did not display social avoidance behavior toward a conspecific mouse (Desbonnet et al., 2012). However, in this social preference test, CSDS mice spent more time with familiar mice. In general, rodents tend to investigate novel same-sex conspecifics (Murugan et al., 2017). However, following exposure to social stress, rodents choose familiar mice (Hammels et al., 2015). This is also in agreement with previous studies where CSDS female mandarin voles (*Microtus mandarinus*) showed avoidance behavior to novel same-sex conspecifics (Wang et al., 2018). This suggests that SD group mice had an increased amount of vigilance when faced with novel mice and even displayed decreased curiosity. In social interaction tests, the SD group showed higher levels of defensive, freeze behavior, aggression, climbing, and exploration compared to controls, which indicates a lack of desire for social interaction. Disruption of natural social behavior is a common symptom of neuropsychiatric disorder (Miyoshi and Morimura, 2010). Previous studies showed that CSDS could induce a deficiency in social interaction (Iniguez et al., 2014; Yin et al., 2015). This result is also consistent with previous reports indicating that CSDS induces social interaction disorder in susceptible mice (Gray et al., 2015; Jin et al., 2015; Huang et al., 2016). Therefore, these findings support the CSDS impaired social preference and induced deficiency in social interaction.

Interestingly, VWR could reverse the impairment of social preference and deficiency in social interaction induced by CSDS. This is consistent with previous studies where exercise before or in the process of CSDS can reverse social avoidance of defeated mice (Otsuka et al., 2015; Mul et al., 2018). The time of VWR in the present study is more similar to the treatment of psychiatric disorders using antidepressants in human. This finding is supported by several lines of evidence, reporting that physical exercise can effectively relieve psychopathological disorders, and the effect of exercise is similar to or more significant than that of a psychological intervention and antidepressant treatment (Blumenthal et al., 2007; Hoffman et al., 2011; Otsuka et al., 2015; Kingston et al., 2018). These results suggest that voluntary exercise is an effective way to treat social interaction disorders induced by social stress.

### Effect of VWR on Levels of VTA TH in CSDS Mice

Chronic social defeat stress also reduced the levels of TH in the VTA. VWR reversed this alteration. The up-regulated levels of TH might increase DA synthesis in the VTA, and increased its release in dopaminergic neuron projection areas, such as the NAc. VTA is the origin of dopaminergic neurons. Tyrosine hydroxylase is a speed-limiting enzyme in dopamine synthesis from tyrosine. Previous findings indicate that dopaminergic neurons in the VTA and their projections to the NAc (but not the mPFC) induced susceptibility to CSDS (Nestler and Carlezon, 2006; Chaudhury et al., 2013). A further report also showed that dopaminergic neurons in the VTA project to the shell of the NAc (Lammel et al., 2011). In addition, optogenetic phasic stimulation of VTA DA neurons also induced a susceptible phenotype in previously resilient TH-Cre mice that have been subjected to CSDS (Chaudhury et al., 2013). The results of the present study agree with these previous findings. Although these results show that VWR reversed the decrease of both TH positive neurons and TH expression in the VTA induced by CSDS, the reversing effect of VWR on the decrease of TH positive neurons in the VTA is more significant than the reversing effect on the decrease of TH expression in the VTA. This discrepancy may be due to methodological differences (immunofluorescence vs. Western blot). The recovery effect of VWR on deficits in social interaction of CSDS mice was related to the levels of TH in the VTA and their projections areas.

### Effect of VWR on Levels of NAc Shell D2R in CSDS Mice

Western blot showed that CSDS decreased D2R protein expression in the NAc shell. No significant difference in D1R expression was observed between control and CSDS groups in the mPFC, NAc core, and shell. These results are consistent with a previous study where CSDS was reported to only alter the dopaminergic projection from VTA to NAc, but had no significant effects on projection from VTA to mPFC (Chaudhury et al., 2013). An increasing number of studies showed that the dysfunction of dopamine and its receptors is an important cause of social defeat stress (Trainor, 2011; Huang et al., 2016). Previous results showed that changes in D1R and D2R expression induced by social stress are inconsistent. For example, D2R density was reported to be elevated in the NAc after single and repeated visible burrow system treatments (a chronic social stress paradigm), but no change in D1R binding was observed (Lucas et al., 2004). Eight weeks of social isolation did not change the D2R expression in the NAc core or shell (Malone et al., 2008). No significant change was observed in adolescent rats in the expression of D2R in the NAc after CSDS (Burke et al., 2011). The results of this study are inconsistent with these previous studies. Although caution needs to be applied in the interpretation of these results, one possible explanation for this inconsistency may be that the levels of the D2R fluctuation depend on different stress. Another possible explanation is that adolescent and adult animals may have different changes of DA receptors in the brain after CSDS. However, the result of the present study is supported by a previous study where chronic passive exposure to aggression was reported to decrease D2R densities in the cortical-accumbal regions (shell of the NAc and cingulate and motor cortices) (Suzuki et al., 2010). Another interesting finding is that 3 week of VWR in mice could increase D2R levels in the NAc shell. Using the running wheel may be a natural reward (Novak et al., 2012), and both genders displayed a strong conditioned place preference associated with running (Basso and Morrell, 2015). Young et al. pointed out that the DA receptor in the NAc plays an important role in the motor motivation underlying voluntary exercise (Park et al., 2016). The results of the present study are inconsistent with a previous report where the activation of dopamine D2/D3 receptors was reported to contribute to the motivation for mice during VWR training (Ebada et al., 2016). The strategy of direct control of neural activity by using designer receptors exclusively activated by designer drugs (DREADD) showed that manipulation of NAc D2R neuron influences the running distance (Zhu et al., 2016). A further possibility suggests that VWR or exercise training increases daily food intake and this increased appetite may possibly induce elevation of D2R expression in NAc. This is possibly also associated with the observed reversal of VWR effects in the present study since it has been reported that increased food intake can also reduce social avoidance behavior induced by mild chronic stress (Otsuka et al., 2015). The results obtained in the present study agree with these previous studies, showing that VWR increased the D2R expression in the NAc shell. An increased running distance may improve the therapeutic effect of voluntary exercise on social interaction disorders. The effects of VWR on social interaction deficits induced by CSDS may be initiated via the upregulation of D2R in the NAc shell.

### D2R Blockage in the NAc Shell Induces Social Interaction Deficits in Mice With SD+VWR

Consistent with the hypothesis of this study, the recovery effect of VWR to CSDS mice on social interaction deficits was blocked via microinjection of the D2R antagonist raclopride into the NAc shell. The results that the VWR reverses CSDS-induced social interaction deficit is consistent with effects reported for antidepressants, which predominantly increase D2R mRNA in the NAc shell (Ainsworth et al., 1998). In addition, activation of D2R in the NAc also has an antidepressant effect (Gershon et al., 2007). Increase of D2R expression in the NAc of adult mice enhances motivation (Trifilieff et al., 2013). However, mice exposed to CSDS displayed a lack of desire for social interaction (Miyoshi and Morimura, 2010; Rosa et al., 2018). Injection of a D2R agonist in the CSDS group might improve deficits in social behavior. Therefore, it can be inferred that impairments in social preference and deficiency in social interaction could be reversed by voluntary exercise via activation of D2R in the NAc shell.

Despite this clear result, this study has several limitations. First, CSDS may affect the level of spontaneous running, and therefore, a control group without CSDS should be included in future research. Second, DA agonist microinjection in the SD group should be included in further investigations to confirm whether exercising decreases CSDS-induced social interaction disorder by increasing D2R in the NAc. Third, although a number of studies reported that CSDS could increase depression-like behaviors in rodents (Garcia-Garcia et al., 2017; Fukumoto et al., 2018), future studies should better test whether VWR could also reverse CSDS-induced depression-like behaviors and whether the 5-HT system is involved in this process.

## Conclusion

In conclusion, this study showed that CSDS can impair social preference and induce a deficiency in social interaction. These effects could be reversed by VWR. Both the TH in the VTA and D2R in the NAc shell may be involved in the processes underlying the reversal of CSDS-induced social interaction deficit mediated by voluntary exercise. This finding suggests that voluntary exercise has positive efficacy in the treatment of CSDS-induced social interaction disorder via alteration of the levels of D2R in the NAc shell. This result indicates that voluntary exercise can treat social interaction disorder induced by CSDS, and its underlying mechanism may be the activation of the DA system. These results suggest that voluntary exercise can be used as a complementary therapy method to treat psychiatric disorders.

## Data Availability

All datasets generated for this study are included in the manuscript.

## Author Contributions

JZ, F-dT, and S-cA designed the experiments. JZ, WY, YY, Q-qG, W-qC, and L-fL performed the experiments. JZ, W-jH, X-nZ, Z-xH, and L-mW analyzed the data and prepared the figures. JZ and F-dT wrote the manuscript. All authors approved the final version of the manuscript.

## Conflict of Interest Statement

The authors declare that the research was conducted in the absence of any commercial or financial relationships that could be construed as a potential conflict of interest.

## Footnotes

1 http://www.jwatcher.ucla.edu/
